# Endoscopist and Patients' Values and Preferences on Artificial Intelligence in Endoscopy: An Intercontinental Opinion Survey by the World Endoscopy Organization

**DOI:** 10.1111/den.70123

**Published:** 2026-02-20

**Authors:** O. F. Ahmad, A. de Groof, A. Ali, P. Bassett, M. Engels, S. Hoogenboom, N. Coelho‐Prabhu, H. Yu, M. Mwachiro, S. Parasa, R. Mansilla Vivar, J. Mushtaq, H. Neumann, S. Thakkar, M. F. Byrne, J. E. van Hooft, T. Yano, Y. Mori

**Affiliations:** ^1^ Division of Surgery and Interventional Science University College London London UK; ^2^ Department of Gastrointestinal Services University College London Hospital London UK; ^3^ Department of Gastroenterology and Hepatology, Amsterdam University Medical Centres Amsterdam the Netherlands; ^4^ Statsconsultancy Ltd Amersham UK; ^5^ Department of Gastroenterology and Hepatology Leiden University Medical Center Leiden the Netherlands; ^6^ Department of Gastroenterology and Hepatology Haga Hospital The Hague the Netherlands; ^7^ Department of Gastroenterology and Hepatology Mayo Clinic Rochester Rochester Minnesota USA; ^8^ Department of Gastroenterology Renmin Hospital of Wuhan University Wuhan China; ^9^ College of Surgeons of East, Central and Southern Africa Arusha Tanzania; ^10^ Swedish Medical Center Seattle Washington USA; ^11^ Digestive Endoscopy Unit Hospital Puerto Montt Puerto Montt Chile; ^12^ Department of Gastroenterology Services Institute of Medical Sciences Lahore Pakistan; ^13^ Department of Endoscopy University Medical Center Mainz Germany; ^14^ Section of Gastroenterology and Hepatology, Department of Medicine West Virginia University Morgantown West Virginia USA; ^15^ Division of Gastroenterology Vancouver General Hospital/University of British Columbia Vancouver British Columbia Canada; ^16^ Department of Gastroenterology and Endoscopy National Cancer Center Hospital East Kashiwa Japan; ^17^ Clinical Effectiveness Research Group University of Oslo Oslo Norway; ^18^ Gastroenterology Section, Department of Transplantation Medicine Oslo University Hospital Oslo Norway; ^19^ Digestive Disease Center Showa University Northern Yokohama Hospital Yokohama Japan

**Keywords:** artificial intelligence, endoscopy, medical liability, patient acceptance of health care, physician‐patient relations

## Abstract

**Background and Aims:**

Artificial intelligence (AI) is increasingly integrated into gastrointestinal (GI) endoscopy, yet limited data exist on how patients and endoscopists perceive its use. This study aimed to evaluate users' values and preferences regarding AI in endoscopy to support effective implementation and inform guideline development.

**Methods:**

As part of the World Endoscopy Organization (WEO) AI committee initiatives, two structured international surveys were conducted—one for patients and one for practicing endoscopists. Thirteen AI‐related statements were presented to patients via an established online platform, while 23 statements were shared with endoscopists through professional networks. Responses were captured using 5‐point Likert scales and analyzed with non‐parametric tests, including subgroup comparisons by age, gender, and endoscopic experience.

**Results:**

A total of 1237 patients and 476 endoscopists participated. Most patients supported AI in image analysis (75.5%) but emphasized the need for endoscopist oversight (92.3%). Among endoscopists, 90.3% believed AI improves endoscopy quality, and 85.3% believed it benefits outcomes. Concerns were raised about liability (47%), operator dependency (34.8%), and procedure time (49%). Most respondents felt primary responsibility for AI‐related errors should rest with the endoscopist. Younger and male patients reported greater trust in AI.

**Conclusions:**

Patients and endoscopists are generally supportive of AI in GI endoscopy, especially as an adjunct to human expertise. However, key concerns—including accountability, trust, and clinical integration—must be addressed to ensure responsible and effective adoption.

## 1 Introduction

In recent years, the number of clinical studies assessing artificial intelligence (AI) systems in endoscopy has grown rapidly, including over 40 randomized trials in colonoscopy [1]. Most of the published research focuses on evaluating AI systems that assist endoscopists with primary detection (computer‐aided detection; CADe; real‐time software to highlight possible lesions) and characterization (computer‐aided diagnosis; CADx; software that provides a classification of a detected lesion) during live procedures [2]. Several AI systems are already commercially available, positioning gastrointestinal (GI) endoscopy as one of the leading areas in the integration of AI in medicine.

However, there is a lack of understanding regarding the opinions on AI from the endoscopic community, who will be the primary users of AI. Preliminary research suggests that while the community recognizes the potential of AI to revolutionize the field of medicine and endoscopy, there are also concerns that could hinder its successful implementation in daily practice [3, 4, 5].

The lack of users' perspective is also a significant concern when developing trustworthy clinical guidelines, as incorporating users' values and preferences is considered a crucial step in the GRADE‐based guideline development process [6]. In fact, the three currently available guidelines on CADe in colonoscopy—by the American Gastroenterology Association, the European Society of Gastrointestinal Endoscopy, and the BMJ—have struggled to incorporate users' perspectives due to a lack of valid studies [7, 8, 9].

Finally, little is known about patient perceptions on the use of AI in endoscopy, even though authorities encourage integration of patients' thoughts in the development of innovative medical interventions to improve patient acceptability and adoption.

Reflecting the need for collecting user feedback, the AI ad hoc Committee of the World Endoscopy Organization (WEO) launched a questionnaire project to improve our understanding with regard to gastrointestinal AI applications. The present manuscript presents the methodology, results, and interpretation of a WEO‐driven large‐scale international endoscopist and patient opinion survey.

## 2 Methods

### 2.1 Study Design

This is a questionnaire study evaluating patient and endoscopist perspectives on the use of AI in endoscopy. The endoscopist survey was carried out between January and April 2023, whereas the patient survey was conducted during July and August 2023. This study was conducted as part of the WEO AI committee activities. The medical ethics committee of the Amsterdam UMC reviewed the study protocol on behalf of the research consortium and ruled that the Medical Research Involving Human Subjects Act (WMO) did not apply to this study. Formal review was therefore waived (METC W22_442).

### 2.2 Patient Questionnaire

The WEO AI committee developed 13 statements (Table 1) on the use of AI in endoscopy, which were distributed to patients using a dedicated online research survey platform (Prolific, London, United Kingdom) that connects researchers with survey participants based on predefined screening criteria.

**TABLE 1:** Patient survey statements.

**Baseline characteristics**
Country of origin
Age
Gender
Gastroenterology patient (Yes/No)
Experience with undergoing endoscopy (Yes/No)
**General AI survey questions**
How much would you say you know about artificial intelligence?
In general, how much do you trust the use of artificial intelligence?
In general, I believe that humans and artificial intelligence can complement each other
**AI in endoscopy survey questions**
I trust the use of artificial intelligence in endoscopy
I believe that an AI system could be better in endoscopic diagnosis than an experienced endoscopist
I would support the use of AI systems that assist physicians in analyzing endoscopic imagery during endoscopy
Even when AI systems are used to assist physicians during endoscopy, the physician should always remain responsible for the decision
The fact that AI systems support physicians in decision‐making worries me
I am afraid that when AI is used, my data might fall into the wrong hands
I am concerned about health care costs in general
It is important that AI systems in endoscopy are cost‐effective
I am concerned about medical liability due to errors associated with AI use
If a medical error occurs where endoscopists use AI as a decision support tool, who do you believe should be held liable?

In evaluating the content of the statements, committee members prioritized aspects most relevant to patients. The statements were initially drafted by the study leads (OFA, AG, and YM), then thoroughly discussed during two online committee meetings, resulting in consensus on the final versions. To limit potential geographical selection bias, the questionnaires were distributed equally to each of the six continents in the World (Africa, Asia, Europe, North America, Oceania, South America) via Prolific. Patients responded to each of the statements by using a 5‐point Likert scale, ranging from strongly disagree (1) to strongly agree (5). Questionnaires were distributed in English. There were no exclusion criteria for participation. Both GI patients and participants without experience in gastroenterology or endoscopy were included.

The patient questionnaire began with a brief introduction, offering participants basic information about endoscopic procedures, along with a basic explanation of CADe and CADx applications, explaining how these might assist endoscopists. This introduction included visual examples of the most common AI applications in endoscopy. The exact text used in this introductory section is included in Data S1. Detailed scientific evidence regarding potential benefits or harms was not provided, although the introductory text stated that an increase in polyp detection has been demonstrated in published studies. At the time of survey creation, major publications demonstrating potential harms of AI in colonoscopy had not been published [10, 11]. The questionnaire consisted of baseline participant information, followed by three questions assessing participants' perceptions of AI in general, and 10 specific questions regarding its use in endoscopy.

### 2.3 Endoscopist Survey

Similar to the patient survey, the committee developed a total of 23 statements (Table 2). The committee also valued what was most relevant to endoscopists when discussing the statements. The endoscopist survey was divided into three sections: (1) baseline participant information (country, age, professional role, type of endoscopic practice, experience level, prior use of AI); (2) general perceptions regarding AI in endoscopy, consisting of 10 statements; (3) specific opinions relating to computer‐aided detection and characterization, consisting of 13 statements. The questionnaires were distributed via the WEO newsletter to its membership and through personal direct email communication via committee members only. Other methods such as social media advertising or on‐site recruitment in endoscopy units were not utilized. Only practicing endoscopists were invited to participate. Endoscopists responded to each of the statements by using a 5‐point Likert scale, ranging from strongly disagree (1) to strongly agree (5). Questionnaires were distributed in English using a web‐based electronic data capture platform (REDCap).

**TABLE 2:** Endoscopist survey statements.

**Baseline characteristics**
Country of origin
Age
Professional role (gastroenterologist, surgeon, general physician, nurse or non‐medical endoscopist, other)
How would you best describe your endoscopy practice (general endoscopist, specialist endoscopist, trainee endoscopist, other)
Years of endoscopic experience
Experience working with AI (Yes/No)
**General AI in endoscopy survey questions**
*Potential clinical benefits*
Endoscopy quality will improve with the use of AI
Endoscopic AI applications will lead to improved patient outcomes
AI will lead to improved efficiency in endoscopy
AI will improve training in endoscopy
AI will lead to operator dependence or de‐skilling of endoscopists
Financial re‐imbursement is important for AI when applied in daily endoscopy practice
AI use in endoscopy will be cost‐effective
AI will negatively impact the physician‐patient relationship
I am concerned about medical liability due to errors associated with AI use
If a medical error occurs where endoscopists use AI as a decision support tool, who do you believe should be held responsible?
**Specific AI survey questions**
*Colonic polyp detection (CADe)*
Endoscopist struggle with detection of colonic polyps
CADe for colonoscopy may help the endoscopist to detect clinically relevant polyps
CADe will lead to unnecessary polyp resections
CADe will lengthen procedural time of colonoscopy
I will likely use a CADe system for polyp detection when available
*Colonic polyp classification (CADx)*
Endoscopists struggle with the differentiation between colorectal hyperplastic polyps and adenomas
I would be willing to leave in a diminutive (≤ 5 mm) rectosigmoid polyp based on optical diagnosis
I feel more comfortable leaving in a diminutive (≤ 5 mm) rectosigmoid hyperplastic polyp if the diagnosis is supported by AI assistance
I will likely use a CADx system for polyp classification when available
*Upper GI neoplasia detection (CADe)*
Endoscopists struggle with detection of upper GI neoplasia
CADe for upper GI neoplasia will help the endoscopist to detect additional neoplastic lesions
CADe will lead to unnecessary additional biopsies and/or resections
I will likely use a CADe system for upper GI neoplasia when available

### 2.4 Outcomes and Statistical Analysis

Results are presented in percentages using Likert‐style ordinal measures. Additional analyses compared responses between different subgroups of patients, based on their gender, age, demographics, and experience with gastroenterology and endoscopy. Comparisons of these outcomes between two groups were made using the Mann–Whitney test, whilst the Kruskal‐Wallis test was used for comparison between three groups. Some questions had no ordering to the responses. These were compared between groups using the Chi‐square test.

## 3 Results

### 3.1 Patient Questionnaire

#### 3.1.1 Baseline Characteristics

In total, 1237 patients completed the questionnaire (> 200 respondents per continent). Approximately half of the respondents were under 30 years old, with an equal distribution of males (47.1%) and females (51.2%). Around a quarter of participants were gastroenterology patients (24.8%) and/or had experience undergoing endoscopy (25.3%). An overview of all baseline characteristics can be found in Table 3.

**TABLE 3:** Baseline characteristics patient survey.

Variable	Category	Number (%)
Age category	18–20	40 (3.2%)
	21–30	614 (49.6%)
	31–40	303 (24.5%)
	41–50	136 (11.0%)
	51–60	88 (7.1%)
	61–70	43 (3.5%)
	71–80	13 (1.1%)
Gender	Female	632 (51.2%)
	Male	582 (47.1%)
	Non‐binary	21 (1.7%)
Gastroenterology patient (current or previous)	No	930 (75.2%)
	Yes	307 (24.8%)
Experience undergoing endoscopy	No	924 (74.7%)
	Yes	313 (25.3%)

#### 3.1.2 Patient Views on AI

Table 4 provides a comprehensive overview of all questionnaire responses. On a 5‐point Likert scale, patients did not exhibit a specific bias for or against AI in general, nor did they report significant knowledge of AI or express notable levels of trust or distrust, although young patients and males indicated to have significantly more knowledge on AI than older patients and females (*p* < 0.001). Subgroup analyses according to patient age and gender are included in Tables S1 and S2, respectively. Most patients (74.3%) indicated that AI and humans can complement each other, while only a small portion reported distrust in the use of AI in endoscopy (15.1%) or were worried about AI supporting physicians (13.3%). The vast majority of respondents supported the use of AI to assist in analyzing endoscopic imagery (75.5%), although the endoscopist should remain responsible for decision‐making (92.3%). Subgroup analyses showed that male respondents had more trust in the use of AI in general (*p* < 0.002) and endoscopy (*p* < 0.001) (Table S2). Only a minority of respondents believed that AI could outperform an experienced endoscopist (27.8%). Patients expressed concerns about healthcare costs (71.8%) and emphasized the importance of AI's cost‐effectiveness (79.9%). Most of the patients believed that medical liability in the event of an error should primarily rest with the health care provider (i.e., endoscopist or hospital; 76.9%), with the manufacturer being a secondary consideration (18%). For all relevant outcomes, there were no statistically significant differences between GI patients or non‐GI patients (Tables S3 and S4).

**TABLE 4:** Summary of patient views on AI.

Statement	Likert scale	Number (%)
Knowledge of AI	1—Very little	46 (3.7%)
	2	212 (17.1%)
	3—Neutral	506 (40.9%)
	4	382 (30.9%)
	5—Significant knowledge	91 (7.4%)
Amount of trust in AI	1—Very little trust	40 (3.2%)
	2	208 (16.8%)
	3—Neutral	536 (43.3%)
	4	376 (30.4%)
	5—Significant trust	77 (6.2%)
Humans and AI can complement each other	1—Strongly disagree	24 (1.9%)
	2—Disagree	73 (5.9%)
	3—Neutral	221 (17.9%)
	4—Agree	481 (38.9%)
	5—Strongly agree	438 (35.4%)
Trust use of AI in endoscopy	1—Strongly disagree	45 (3.6%)
	2—Disagree	142 (11.5%)
	3—Neutral	373 (30.2%)
	4—Agree	491 (39.7%)
	5—Strongly agree	186 (15.0%)
AI better than experienced endoscopist	1—Strongly disagree	143 (11.6%)
	2—Disagree	329 (26.6%)
	3—Neutral	421 (34.0%)
	4—Agree	236 (19.1%)
	5—Strongly agree	108 (8.7%)
Support use AI to assist in analyzing endoscopic imagery	1—Strongly disagree	26 (2.1%)
	2—Disagree	67 (5.4%)
	3—Neutral	210 (17.0%)
	4—Agree	502 (40.6%)
	5—Strongly agree	432 (34.9%)
Physician should remain responsible	1—Strongly disagree	7 (0.6%)
	2—Disagree	11 (0.9%)
	3—Neutral	77 (6.2%)
	4—Agree	257 (20.8%)
	5—Strongly agree	885 (71.5%)
Worried by AI supporting physicians	1—Strongly disagree	280 (22.6%)
	2—Disagree	532 (43.0%)
	3—Neutral	260 (21.0%)
	4—Agree	114 (9.2%)
	5—Strongly agree	51 (4.1%)
Afraid data would fall into wrong hands	1—Strongly disagree	247 (20.0%)
	2—Disagree	361 (29.2%)
	3—Neutral	279 (22.6%)
	4—Agree	215 (17.4%)
	5—Strongly agree	135 (10.9%)
Concerned about health costs	1—Strongly disagree	67 (5.4%)
	2—Disagree	107 (8.7%)
	3—Neutral	174 (14.1%)
	4—Agree	379 (30.6%)
	5—Strongly agree	510 (41.2%)
Important AI is cost‐effective	1—Strongly disagree	10 (0.8%)
	2—Disagree	30 (2.4%)
	3—Neutral	209 (16.9%)
	4—Agree	467 (37.8%)
	5—Strongly agree	521 (42.1%)
Concerned about medical liability	1—Strongly disagree	54 (4.4%)
	2—Disagree	193 (15.6%)
	3—Neutral	364 (29.4%)
	4—Agree	373 (30.2%)
	5—Strongly agree	253 (20.5%)
Liability for medical error	AI manufacturer	222 (18.0%)
	Endoscopist	560 (45.3%)
	Hospital	391 (31.6%)
	Insurance company	41 (3.3%)
	Other	23 (1.9%)

### 3.2 Endoscopist Questionnaire

#### 3.2.1 Baseline Characteristics

In total, 476 endoscopists completed the survey. Almost a third were aged 40 years or under. The majority were gastroenterologists (81.9%) with over half working in specialist endoscopy (54.1%). Approaching half (46%) had > 15 years of endoscopy experience. Over a third had experience with AI in clinical practice.

The majority of responses were from Asia (32.7%), Europe (22.5%), South America (22.9%), and North America (13.0%). An overview of endoscopist characteristics can be found in Table 5.

**TABLE 5:** Summary of baseline characteristics of endoscopist survey.

Variable	Category	Number (%)
Age category	20–30	21 (4.5%)
	31–40	119 (25.2%)
	41–50	149 (31.6%)
	51–60	97 (20.6%)
	61–70	74 (15.7%)
	71–80	12 (2.5%)
Profession	Gastroenterologist	388 (81.9%)
	Surgeon	56 (11.8%)
	General surgeon	10 (2.1%)
	Nurse	8 (1.7%)
	Other	12 (2.5%)
Endoscopy practice	General endoscopist	169 (35.6%)
	Specialist endoscopist	257 (54.1%)
	Trainee endoscopist	47 (9.9%)
	Other	2 (0.4%)
Endoscopic experience	0–5 years	90 (19.0%)
	6–10 years	87 (18.3%)
	11–15 years	79 (16.6%)
	16–20 years	57 (12.0%)
	> 20 years	162 (34.1%)
Experience of AI in clinical practice	No	294 (61.9%)
	Yes	181 (38.1%)

#### 3.2.2 General Views of AI in Endoscopy

Tables 6 and 7 provide a comprehensive overview of all questionnaire responses. Approximately 90% of endoscopists thought that AI improved the quality of endoscopy, with around 85% suggesting that it improved patient outcomes. There were also positive views on improved efficiency and that AI improved training, with over 80% agreeing (or strongly agreeing) with these statements.

**TABLE 6:** Summary of endoscopist views on AI.

Variable	Category	Number (%)
Endoscopy quality improved	Strongly agree	185 (39.0%)
	Agree	244 (51.5%)
	Neutral	40 (8.4%)
	Disagree	5 (1.1%)
	Strongly disagree	0 (0.0%)
Improved patient outcome	Strongly agree	151 (31.8%)
	Agree	257 (54.1%)
	Neutral	60 (12.6%)
	Disagree	6 (1.3%)
	Strongly disagree	1 (0.2%)
Improved efficiency	Strongly agree	168 (35.5%)
	Agree	240 (50.7%)
	Neutral	54 (11.4%)
	Disagree	11 (2.3%)
	Strongly disagree	0 (0.0%)
Improve training	Strongly agree	180 (38.0%)
	Agree	204 (43.0%)
	Neutral	67 (14.1%)
	Disagree	21 (4.4%)
	Strongly disagree	2 (0.4%)
Operator dependence/de‐skilling	Strongly agree	42 (8.8%)
	Agree	135 (28.4%)
	Neutral	173 (36.4%)
	Disagree	113 (23.8%)
	Strongly disagree	12 (2.5%)
Financial reimbursement important	Strongly agree	122 (25.7%)
	Agree	219 (46.2%)
	Neutral	109 (23.0%)
	Disagree	22 (4.6%)
	Strongly disagree	2 (0.4%)
Cost effective	Strongly agree	69 (14.7%)
	Agree	192 (40.7%)
	Neutral	151 (32.0%)
	Disagree	57 (12.1%)
	Strongly disagree	3 (0.6%)
Negatively impact patient relationship	Strongly agree	15 (3.2%)
	Agree	44 (9.3%)
	Neutral	107 (22.6%)
	Disagree	250 (52.7%)
	Strongly disagree	58 (12.2%)
Concerned about medical liability	Strongly agree	49 (10.3%)
	Agree	175 (36.7%)
	Neutral	129 (27.2%)
	Disagree	109 (23.0%)
	Strongly disagree	12 (2.5%)
Liability for medical error	Endoscopist	267 (56.3%)
	Hospital	37 (7.8%)
	AI manufacturer	105 (22.2%)
	Insurance company	17 (3.6%)
	Other	48 (10.1%)

**TABLE 7:** Summary of views on specific AI applications.

Variable	Category	Number (%)
Endoscopists struggle with detection colonic polyps	Strongly agree	70 (14.8%)
	Agree	232 (49.0%)
	Neutral	76 (16.0%)
	Disagree	88 (18.6%)
	Strongly disagree	8 (1.7%)
CADe helps detect clinically relevant polyps	Strongly agree	113 (23.9%)
	Agree	291 (61.7%)
	Neutral	44 (9.3%)
	Disagree	23 (4.9%)
	Strongly disagree	1 (0.2%)
CADe leads to unnecessary polyp resections	Strongly agree	36 (7.6%)
	Agree	156 (33.0%)
	Neutral	124 (26.2%)
	Disagree	141 (29.8%)
	Strongly disagree	16 (3.4%)
CADe lengthens procedure times	Strongly agree	40 (8.5%)
	Agree	190 (40.2%)
	Neutral	118 (25.0%)
	Disagree	118 (25.0%)
	Strongly disagree	7 (1.5%)
Use CADe when available	Strongly agree	134 (28.4%)
	Agree	261 (55.3%)
	Neutral	59 (12.5%)
	Disagree	15 (3.2%)
	Strongly disagree	3 (0.6%)
Endoscopists struggle differentiate	Strongly agree	69 (14.6%)
Hyperplastic polyps and adenomas	Agree	239 (50.5%)
	Neutral	75 (15.9%)
	Disagree	83 (17.6%)
	Strongly disagree	7 (1.5%)
Willing leave diminutive rectosigmoid polyp	Strongly agree	82 (17.3%)
	Agree	242 (51.1%)
	Neutral	58 (12.2%)
	Disagree	79 (16.7%)
	Strongly disagree	13 (2.7%)
More comfortable leaving polyp if supported by AI	Strongly agree	96 (20.3%)
	Agree	225 (47.6%)
	Neutral	87 (18.4%)
	Disagree	56 (11.8%)
	Strongly disagree	9 (1.9%)
Use CADx system when available	Strongly agree	128 (27.1%)
	Agree	285 (60.3%)
	Neutral	50 (10.6%)
	Disagree	9 (1.9%)
	Strongly disagree	1 (0.2%)
Endoscopists struggle with detection upper GI neoplasia	Strongly agree	141 (29.8%)
	Agree	269 (56.9%)
	Neutral	53 (11.2%)
	Disagree	9 (1.9%)
	Strongly disagree	1 (0.2%)
CADe helpful for upper GI neoplasia	Strongly agree	141 (29.8%)
	Agree	269 (56.9%)
	Neutral	53 (11.2%)
	Disagree	9 (1.9%)
	Strongly disagree	1 (0.2%)
CADe leads to additional biopsies in upper GI tract	Strongly agree	36 (7.6%)
	Agree	147 (31.1%)
	Neutral	135 (28.5%)
	Disagree	143 (30.2%)
	Strongly disagree	12 (2.5%)
Use CADe for GI neoplasia when available	Strongly agree	172 (36.4%)
	Agree	248 (52.4%)
	Neutral	48 (10.2%)
	Disagree	5 (1.1%)
	Strongly disagree	0 (0.0%)

There were more mixed views on whether operators were dependent on AI. Over a third agreed that operators were dependent, but around 30% disagreed. The majority thought financial reimbursement was important (71.9%) and over half (55.4%) felt that AI was cost‐effective. Only 13% felt AI would negatively impact the physician‐patient relationship. Almost half (47%) were concerned about liability. When directly comparing responses from endoscopists and patients, endoscopists were significantly more likely to attribute responsibility to themselves (56%) than patients were (45%; *p* < 0.001). Conversely, patients were substantially more likely to hold the hospital responsible, with nearly one‐third (32%) giving this view, compared to only 8% of endoscopists (*p* < 0.001).

#### 3.2.3 Views on Specific AI Applications

Over 80% of endoscopists felt that CADe helped with polyp detection. There were mixed views on whether CADe led to unnecessary polyp resections, with a similar proportion agreeing and disagreeing with this statement. Almost half (49%) felt CADe lengthened procedure times. However, over 80% would use CADe when available.

Around 65% of endoscopists believed that endoscopists struggled to differentiate between hyperplastic polyps and adenomas. A similar proportion were willing to leave diminutive rectosigmoid polyps in situ. Around two‐thirds of patients (68%) felt more comfortable leaving a polyp if this decision was supported by AI. The majority (87%) would use CADx if available.

Over 85% felt that endoscopists struggle with detecting upper GI neoplasia, and a similar proportion felt that CADe would be helpful for this type of neoplasia. There were mixed views as to whether CADe would lead to additional biopsies in the upper GI tract. Almost 40% agreed with this statement, but around a third disagreed.

Additional results including subgroup analyses are described in Tables S5–S9.

## 4 Discussion

This large‐scale, international survey conducted by the WEO provides valuable insights into patient and endoscopist perspectives on the use of AI in gastrointestinal endoscopy. The findings reveal generally positive attitudes toward AI among both stakeholder groups, with high levels of interest in its clinical utility and recognition of its potential to enhance diagnostic accuracy and efficiency.

Importantly, the results also highlight critical concerns and divergent opinions that must be addressed to ensure successful and responsible integration of AI into clinical endoscopic practice, including during the guideline development process.

A key finding from both surveys is the widespread support for AI‐assisted technologies, particularly in enhancing diagnostic capabilities such as polyp detection and characterization. Over 80% of endoscopists supported the use of CADe and CADx, reflecting alignment with previous studies demonstrating improved adenoma detection rates (ADR) when using AI‐based tools. Likewise, patients generally supported AI applications, especially when positioned as complementary to human expertise. However, over 90% of patients emphasized that final decision‐making should remain the responsibility of the endoscopist, highlighting the importance of human oversight. This aligns with current endoscopic practice, where AI tools typically act as second readers. These findings underscore an essential point: while stakeholders appreciate the benefits of AI, trust and perceived accountability remain crucial for its acceptance. Both patients and endoscopists expressed concerns about medical liability. Endoscopists were significantly more likely to attribute responsibility to themselves than to patients. Whilst patients were more likely to hold the hospital responsible. These findings demonstrate notable divergence in how liability is perceived with AI‐assisted endoscopy and a possible accountability gap. Endoscopists may view liability as personal, holding themselves as final decision makers and possibly internalize responsibility. Conversely, patients often conceptualize responsibility at the institutional level. This suggests that patients may place greater emphasis on institutional governance to ensure that AI is implemented safely. A notable proportion also identified manufacturers as potentially liable. These concerns are echoed in legal and ethical discussions surrounding AI in medicine, where the distribution of accountability remains unresolved. This ambiguity may pose a barrier to adoption unless explicitly addressed in policy frameworks and clinical guidelines [12].

Interestingly, the surveys revealed generational and gender‐based variations in AI familiarity and trust. Younger participants and male respondents reported greater trust in AI. Notably, older participants were more likely to believe that AI could outperform an experienced endoscopist. This seems counterintuitive since older participants are generally considered to have lower digital literacy. However, this may reflect a greater awareness of human fallibility in medicine along with prevalent media portrayals of ‘superhuman’ AI leading to technological overconfidence in this demographic. Meanwhile, younger participants may be more aware of limitations, particularly with greater exposure to AI tools. The introductory explanatory text may have also influenced responses; however, survey methodology research indicates that respondents vary substantially in their engagement with such text [13, 14]. Future implementation strategies should consider diverse interventions tailored to different demographic groups to ensure equitable engagement with AI tools: for example, materials for older adults may need to highlight existing scientific evidence to mitigate technological overconfidence, emphasizing current limitations and the assistive nature of AI tools in endoscopy. Notably, GI patients did not significantly differ from non‐GI participants in their views on AI, suggesting that attitudes may be more influenced by general perceptions of technology than by direct clinical experience.

From the endoscopist's perspective, AI is widely viewed as a tool to improve the quality of endoscopy, efficiency, and training. Yet, a considerable proportion of endoscopists expressed concerns about operator dependency, increased procedure times, and potential overdiagnosis—particularly with CADe potentially flagging diminutive or non‐neoplastic lesions. A recently published observational study in fact showed potential risk of de‐skilling after long‐term exposure to CADe, probably due to endoscopists' overdependency on the technology [15].

The mixed responses regarding CADx applications, particularly in differentiating hyperplastic from adenomatous polyps and in managing diminutive polyps, highlight a current gap in clinical confidence and infrastructure readiness. Although many endoscopists support the leave in situ strategy when backed up by AI, actual clinical adoption may lag without robust validation studies, regulatory approval, and medico‐legal clarity. Similarly, the strong perceived need for AI in upper GI neoplasia detection reflects the complexity of these lesions and the potential for AI to support diagnosis in less standardized or lower‐volume contexts.

This study has several strengths in comparison with the previously published questionnaire studies in the field [3, 4, 5]. The main advantages include its intercontinental reach, large sample size, and inclusion of both patients and practitioners. It is among the first to systematically document and compare the views of these key stakeholders in the domain of AI in endoscopy, providing a necessary foundation for future guideline development that aligns with the GRADE approach, which emphasizes integrating the values and preferences of the users.

Nevertheless, the study has limitations. First, responses were collected using English‐language online platforms, which may limit generalizability to non‐English‐speaking or digitally underserved populations. The use of an online research survey platform will also have contributed to a relatively young respondent demographic in the patient survey. The relatively young patient cohort, along with potential selection bias among innovation‐oriented endoscopists, may have influenced the results in favor of AI. This younger cohort of participants may not reflect the typical age distribution of patients undergoing GI endoscopy, which may affect the generalizability of the findings.

Furthermore, only 25% of respondents were GI patients and/or had prior experience with endoscopy. However, there were no statistically significant differences between GI patients and non‐GI patients, suggesting that the results may be generalizable to the broader GI patient population.

Second, while we aimed for global representation, regional differences in AI exposure, clinical infrastructure, healthcare system factors, and policy may influence attitudes in ways not fully captured by the survey. Especially, we lack the number of responding endoscopists in underserved areas such as Africa, which could hinder generalization of our message. Third, selection bias may have occurred, particularly among endoscopists with an interest in innovation, potentially skewing results toward more favorable views. Fourth, we aimed to provide basic, neutral information on AI in endoscopy in the introduction to the survey; however, this information may have influenced participants' responses. Evidence regarding potential disadvantages of using AI software such as increased non‐neoplastic polyp resections or increased surveillance burden with CADe use was not included; however, at the time of survey creation, major publications highlighting potential harms of CADe had not been published [10, 11]. Fifth, in the patient survey, we did not collect specific information on regional or situational factors such as health insurance systems and coverage. Similarly, endoscopist participants were not asked about their practice setting or reimbursement structure. It was also not possible to calculate non‐response rates for the survey; to maximize participation, the survey invitation link was distributed widely via a newsletter and personal email communication, encouraging dissemination among endoscopists in a non‐identifiable manner. Future studies should aim to include methods to quantify non‐response rates. We acknowledge that these factors may have influenced participant responses, and future studies should explore these aspects in greater detail. Finally, the absence of patient involvement in the development of the questionnaire is a notable limitation, as user participation is encouraged in research addressing values and preferences. This is especially relevant for participants who have not undergone endoscopic procedures in the past, where more detailed explanatory material could improve understanding. The absence of psychometric validation of our survey instrument could also mean that our responses contain interpretive variability. Accordingly, our results should be reviewed as descriptive rather than definitive measures of attitudes, and future research should incorporate structured psychometric development and validation.

This study highlights several considerations for implementing AI in GI endoscopy, taking into account end‐user preferences and values to inform future guidelines [16]. Both endoscopists and patients supported assistive AI, emphasizing the need for clear guidance on oversight, whilst concerns about liability indicate the importance of medico‐legal frameworks for AI‐related errors. Variations in trust and expectations across demographic groups suggested that tailored communication strategies may be required for optimal AI use. Finally, addressing concerns about increased procedural time, cost‐effectiveness, and financial reimbursement appears important for adoption.

In conclusion, this large‐scale international survey highlights broadly positive attitudes toward AI in GI endoscopy among both patients and endoscopists. While AI is seen as a valuable tool to enhance diagnostic accuracy and efficiency, concerns around trust, accountability, and over‐reliance remain significant. These findings emphasize the need for transparent guidelines, robust validation, and inclusive implementation strategies. Addressing legal, ethical, and demographic challenges will be essential to ensure responsible, equitable adoption of AI in clinical practice.

## Author Contributions

O.F.A., A.G., Y.M.: conception, design, and drafting of the article. All authors: analysis and interpretation of the data, critical revision of the article for important intellectual content, and final approval of the article.

## Funding

Y.M.—European Commission (No. 101057099) and Japan Society of Promotion of Science (No. 22H03357). O.F.A.—European Commission (No. 101057099).

## Ethics Statement

The medical ethics committee of the Amsterdam UMC reviewed the study protocol on behalf of the research consortium and ruled that the Medical Research Involving Human Subjects Act (WMO) did not apply to this study. Formal review was therefore waived (METC W22_442). Informed consent was obtained via the online survey platform.

## Conflicts of Interest

Y.M.: Olympus Corp. (consultancy, lecture fees, device loan); Cybernet System Corp. (loyalty). O.F.A.: Olympus Corp. (Consultancy, Speaker Fees), Odin Vision Ltd. (Consultancy), Medtronic (Consultancy), Boston Scientific (Consultancy), Norgine (Consultancy, Speaker Fees). N.C.‐P.: Boston Scientific (Speaker fees), Iterative Health (Consultancy). J.E.H.: Boston Scientific (Speaker fees), Cook Medical (Speaker fees), Fuji Film (Speaker fees), Falk (Speaker fee), Olympus Medical (Consultancy). M.F.B.: Shareholder, Dova Health Intelligence. R.M.V.: Pentax Comp (Speaker Fees). T.Y.: Olympus (consultancy, lecture fees, research fund), Fujifilm (lecture fees, research fund), HOYA PENTAX (research fund). The remaining authors declare no conflicts of interest.

## Supporting information


**Table S1:** Comparisons between age groups in patient survey.
**Table S2:** Comparisons between genders in patient survey.
**Table S3:** Comparisons between gastroenterology patients and non‐gastroenterology patients in patient survey.
**Table S4:** Comparisons between patients with and without endoscopy experience in patient survey.
**Table S5:** Comparisons of endoscopist general views on AI by type of endoscopy practice.
**Table S6:** Comparisons of specific AI applications questions by type of endoscopy practice.
**Table S7:** Comparisons of endoscopist general views on AI by years of endoscopic experience.
**Table S8:** Comparisons of specific AI applications questions by years of endoscopic experience.
**Table S9:** Comparisons of specific AI applications questions between those with and without practical AI experience.
