# Supplementary material for: Endoscopist and Patients' Values and Preferences on Artificial Intelligence in Endoscopy: An Intercontinental Opinion Survey by the World Endoscopy Organization
Source: Dig Endosc. 2026 Feb 20;38(2):e70123. doi: 10.1111/den.70123 (PMC12921463; doi:10.1111/den.70123)
Supplement: Supplementary file 1 — Table S1: Comparisons between age groups in patient survey. Table S2: Comparisons between genders in patient survey. Table S3: Comparisons between gastroenterology patients and non‐gastroenterology patients in patient survey. Table S4: Comparisons between patients with and without endoscopy experience in patient survey. Table S5: Comparisons of endoscopist general views on AI by type of endoscopy practice. Table S6: Comparisons of specific AI applications questions by type of endoscopy practice. Table S7: Comparisons of endoscopist general views on AI by years of endoscopic experience. Table S8: Comparisons of specific AI applications questions by years of endoscopic experience. Table S9: Comparisons of specific AI applications questions between those with and without practical AI experience. [file DEN-38-0-s001.docx]

**SUPPLEMENTARY MATERIALS**

**INFORMATION PROVIDED TO PATIENTS PRIOR TO SURVEY PARTICIPATION**

*Thank you for agreeing to participate in our survey on artificial intelligence (AI) in endoscopy. This survey has been organised by the World Endoscopy Organisation (WEO) AI comittee. We are aiming to obtain patient opinions on the use of AI in endoscopy. This survey should take approximately 10 minutes to complete.  Anonymous responses are stored.*

*Endoscopy is a test to look inside the body. A thin tube with a camera, called an endoscope, is passed into the body through an opening such as the mouth.*

*The doctor performing the endoscopy usually performs this to look for abnormalities in the gastrointestinal tract (food pipe, stomach or bowel). This includes looking for cancer or pre-cancerous abnormalities (abnormal areas that might turn into cancer in the future if left untreated).*

*Endoscopy is highly operator dependent, meaning the ability to find abnormalities in the gastrointestinal tract (food pipe, stomach or bowel) varies according to the doctor performing the test. Also, the ability to analyse and understand what the appearances of the abnormalities might mean when looked at during the endoscopy test can vary according the person performing the test. This means that abnormalities might be analysed as being benign (non-cancerous), pre-cancerous (abnormal changes before cancer develops) or cancer depending on the interpretation of the image during the test.*

*More recently, computer software using artificial intelligence has been designed for use during the endoscopy test. The software can help detect abnormalities on the endoscopy screen during the endoscopy and also help with interpretation/analysis of areas that might be abnormal.*

*The computer software using artificial intelligence is developed and trained by providing many, often millions, of images (data) from previous endoscopy tests so that the software can learn to detect (find) and diagnose (analyse) abnormalities.*

*The computer software detection systems, for example shown in the first picture below, can place a coloured box or another type of visual alert during the test around any suspected abnormal area, to highlight this to the doctor performing the endoscopy test. This might reduce the possibility of the doctor missing abnormal areas during the test. Many scientific studies have already been published demonstrating an increase in number of abnormal polyps (growths on the lining of the bowel) during endoscopy when AI software is used for example.
The computer software diagnosis systems, for example shown in the second picture below, can also help assist the endoscopist to understand if an area that is found is abnormal or normal. For example, whether the area is benign (non-cancerous), pre-cancerous (changes before cancer develops) or cancer. This might help the endoscopist decide if treatment is needed such as a biopsy or removal of the area.*

**SUPPLEMENTARY TABLES**

*Supplementary Table 1: Comparisons between age groups in patient survey*

| *Variable* | *Category* | *Age ≤ 30*  *n (%)* | *Age 31-50*  *n (%)* | *Age > 50*  *n (%)* | *P-value* |
| --- | --- | --- | --- | --- | --- |
|  |  |  |  |  |  |
| *Knowledge of AI* | *Little* | *96 (15.0%)* | *83 (19.8%)* | *33 (25.0%)* | ***<0.001*** |
|  | *Neutral* | *252 (39.4%)* | *192 (45.8%)* | *62 (47.0%)* |  |
|  | *Lot* | *292 (45.6%)* | *144 (34.4%)* | *37 (28.0%)* |  |
|  |  |  |  |  |  |
| *Trust in AI* | *Little trust* | *124 (19.0%)* | *99 (22.6%)* | *25 (17.4%)* | *0.17* |
|  | *Neutral* | *280 (42.8%)* | *192 (43.7%)* | *64 (44.4%)* |  |
|  | *Significant* | *250 (38.2%)* | *148 (33.7%)* | *55 (38.2%)* |  |
|  |  |  |  |  |  |
| *Humans and AI* | *Disagree* | *52 (8.0%)* | *37 (8.4%)* | *8 (5.6%)* | *0.23* |
| *can complement* | *Neutral* | *107 (16.4%)* | *83 (18.9%)* | *31 (21.5%)* |  |
| *each other* | *Agree* | *495 (75.7%)* | *319 (72.7%)* | *105 (72.9%)* |  |
|  |  |  |  |  |  |
| *Trust use of AI in* | *Disagree* | *102 (15.6%)* | *66 (15.0%)* | *19 (13.2%)* | *0.87* |
| *endoscopy* | *Neutral* | *206 (31.5%)* | *121 (27.6%)* | *46 (31.9%)* |  |
|  | *Agree* | *346 (52.9%)* | *252 (57.4%)* | *79 (54.9%)* |  |
|  |  |  |  |  |  |
| *AI better than* | *Disagree* | *263 (40.2%)* | *165 (37.6%)* | *44 (30.6%)* | ***0.02*** |
| *experienced* | *Neutral* | *224 (34.3%)* | *148 (33.7%)* | *49 (34.0%)* |  |
| *endoscopist* | *Agree* | *167 (25.5%)* | *126 (28.7%)* | *51 (35.4%)* |  |
|  |  |  |  |  |  |
| *Support AI in* | *Disagree* | *48 (7.3%)* | *35 (8.0%)* | *10 (6.9%)* | *0.22* |
| *analysing* | *Neutral* | *112 (17.1%)* | *81 (18.5%)* | *17 (11.8%)* |  |
| *imagery* | *Agree* | *494 (75.5%)* | *323 73.6%)* | *117 (81.3%)* |  |
|  |  |  |  |  |  |
| *Physician should* | *Disagree* | *11 (1.7%)* | *6 (1.4%)* | *1 (0.7%)* | *0.21* |
| *remain* | *Neutral* | *46 (7.0%)* | *23 (5.2%)* | *8 (5.6%)* |  |
| *responsible* | *Agree* | *597 (91.3%)* | *410 (93.4%)* | *135 (93.8%)* |  |
|  |  |  |  |  |  |
| *Worried by AI* | *Disagree* | *426 (65.1%)* | *286 (65.2%)* | *100 (69.4%)* | *0.50* |
| *supporting* | *Neutral* | *141 (21.6%)* | *93 (21.2%)* | *26 (18.1%)* |  |
| *physicians* | *Agree* | *87 (13.3%)* | *60 (13.7%)* | *18 (12.5%)* |  |
|  |  |  |  |  |  |
| *Afraid data fall* | *Disagree* | *302 (46.2%)* | *211 (48.1%)* | *95 (66.0%)* | ***<0.001*** |
| *into wrong hands* | *Neutral* | *141 (21.6%)* | *109 (24.8%)* | *29 (20.1%)* |  |
|  | *Agree* | *211 (32.3%)* | *119 (27.1%)* | *20 (13.9%)* |  |
|  |  |  |  |  |  |
| *Concerned about* | *Disagree* | *86 (13.2%)* | *72 (16.4%)* | *16 (11.1%)* | *0.18* |
| *health costs* | *Neutral* | *83 (12.7%)* | *66 (15.0%)* | *25 (17.4%)* |  |
|  | *Agree* | *485 (74.2%)* | *301 (68.6%)* | *103 (71.5%)* |  |
|  |  |  |  |  |  |
| *Important AI is* | *Disagree* | *21 (3.2%)* | *12 (2.7%)* | *7 (4.9%)* | *0.91* |
| *cost effective* | *Neutral* | *111 (17.0%)* | *78 (17.8%)* | *20 (13.9%)* |  |
|  | *Agree* | *522 (79.8%)* | *349 (79.5%)* | *117 (81.3%)* |  |
|  |  |  |  |  |  |
| *Concerned about* | *Disagree* | *103 (15.8%)* | *101 (23.0%)* | *43 (29.9%)* | ***<0.001*** |
| *medical liability* | *Neutral* | *178 (27.2%)* | *133 (30.3%)* | *53 (36.8%)* |  |
|  | *Agree* | *373 (57.0%)* | *205 (46.7%)* | *48 (33.3%)* |  |
|  |  |  |  |  |  |
| *Liability for* | *Manufacturer* | *136 (20.8%)* | *63 (14.4%)* | *23 (16.0%)* | ***0.01*** |
| *medical error* | *Endoscopist* | *285 (43.6%)* | *201 (45.8%)* | *74 (51.4%)* |  |
|  | *Hospital* | *204 (31.2%)* | *153 (34.9%)* | *34 (31.6%)* |  |
|  | *Insurance* | *18 (2.8%)* | *16 (3.6%)* | *7 (4.9%)* |  |
|  | *Other* | *11 (1.7%)* | *6 (1.4%)* | *6 (4.2%)* |  |
|  |  |  |  |  |  |

*Supplementary Table 2: Comparisons between genders in patient survey*

| Variable | Category | Female  n (%) | Male  n (%) | P-value |
| --- | --- | --- | --- | --- |
|  |  |  |  |  |
| Knowledge of AI | Little | 140 (23.4%) | 70 (12.3%) | **<0.001** |
|  | Neutral | 262 (43.8%) | 237 (41.6%) |  |
|  | Lot | 196 (32.8%) | 263 (46.1%) |  |
|  |  |  |  |  |
| Trust in AI | Little trust | 139 (22.0%) | 102 (17.5%) | **0.002** |
|  | Neutral | 285 (45.1%) | 243 (41.8%) |  |
|  | Significant | 208 (32.9%) | 237 (40.7%) |  |
|  |  |  |  |  |
| Humans and AI can | Disagree | 68 (10.8%) | 25 (4.3%) | **<0.001** |
| complement each | Neutral | 141 (22.3%) | 76 (13.1%) |  |
| other | Agree | 423 (66.9%) | 481 (82.7%) |  |
|  |  |  |  |  |
| Trust use of AI in | Disagree | 120 (19.0%) | 64 (11.0%) | **<0.001** |
| endoscopy | Neutral | 199 (31.5%) | 166 (28.5%) |  |
|  | Agree | 313 (49.5%) | 353 (60.5%) |  |
|  |  |  |  |  |
| AI better than | Disagree | 263 (41.6%) | 194 (33.3%) | **<0.001** |
| experienced | Neutral | 224 (35.4%) | 191 (32.8%) |  |
| endoscopist | Agree | 145 (22.9%) | 197 (33.9%) |  |
|  |  |  |  |  |
| Support AI in | Disagree | 63 (10.0%) | 27 (4.6%) | **<0.001** |
| analysing imagery | Neutral | 131 (20.7%) | 75 (12.9%) |  |
|  | Agree | 438 (69.3%) | 480 (82.5%) |  |
|  |  |  |  |  |
| Physician should | Disagree | 10 (1.6%) | 8 (1.4%) | 0.08 |
| remain responsible | Neutral | 34 (5.4%) | 42 (7.2%) |  |
|  | Agree | 588 (91.3%) | 532 (91.4%) |  |
|  |  |  |  |  |
| Worried by AI | Disagree | 389 (61.6%) | 411 (70.6%) | **0.002** |
| supporting physicians | Neutral | 148 (23.4%) | 107 (18.4%) |  |
|  | Agree | 95 (15.0%) | 64 (11.0%) |  |
|  |  |  |  |  |
| Afraid data fall | Disagree | 312 (49.4%) | 286 (49.1%) | 0.64 |
| into wrong hands | Neutral | 145 (22.9%) | 129 (22.2%) |  |
|  | Agree | 175 (27.7%) | 167 (28.7%) |  |
|  |  |  |  |  |
| Concerned about | Disagree | 105 (16.6%) | 68 (11.7%) | 0.66 |
| health costs | Neutral | 76 (12.0%) | 95 (16.3%) |  |
|  | Agree | 451 (71.4%) | 419 (72.0%) |  |
|  |  |  |  |  |
| Important AI is cost | Disagree | 20 (3.2%) | 17 (2.9%) | 0.61 |
| effective | Neutral | 123 (19.5%) | 83 (14.3%) |  |
|  | Agree | 489 (77.4%) | 482 (82.8%) |  |
|  |  |  |  |  |
| Concerned about | Disagree | 125 (19.8%) | 120 (20.6%) | 0.06 |
| medical liability | Neutral | 176 (27.9%) | 186 (32.0%) |  |
|  | Agree | 331 (55.4%) | 276 (47.4%) |  |
|  |  |  |  |  |
| Liability for medical | Manufacturer | 127 (20.1%) | 86 (14.8%) | 0.07 |
| error | Endoscopist | 285 (45.1%) | 267 (45.9%) |  |
|  | Hospital | 191 (30.2%) | 196 (33.7%) |  |
|  | Insurance | 16 (2.5%) | 24 (4.1%) |  |
|  | Other | 13 (2.1%) | 9 (1.6%) |  |
|  |  |  |  |  |

*Supplementary Table 3: Comparisons between gastroenterology patients and non-gastroenterology patients in patient survey*

| Variable | Category | Non-GI patient  n (%) | GI patient  n (%) | P-value |
| --- | --- | --- | --- | --- |
|  |  |  |  |  |
| Knowledge of AI | Little | 165 (18.4%) | 47 (16.0%) | 0.61 |
|  | Neutral | 382 (42.6%) | 124 (42.2%) |  |
|  | Lot | 350 (39.0%) | 123 (41.8%) |  |
|  |  |  |  |  |
| Trust in AI | Little trust | 197 (21.2%) | 51 (16.6%) | **0.02** |
|  | Neutral | 407 (43.8%) | 129 (42.0%) |  |
|  | Significant | 326 (35.1%) | 127 (41.4%) |  |
|  |  |  |  |  |
| Humans and AI can | Disagree | 79 (8.5%) | 18 (5.9%) | 0.20 |
| complement each | Neutral | 165 (17.7%) | 56 (18.2%) |  |
| other | Agree | 686 (73.8%) | 233 (75.9%) |  |
|  |  |  |  |  |
| Trust use of AI in | Disagree | 146 (15.7%) | 41 (13.4%) | 0.30 |
| endoscopy | Neutral | 284 (30.5%) | 89 (29.0%) |  |
|  | Agree | 500 (53.8%) | 177 (57.7%) |  |
|  |  |  |  |  |
| AI better than | Disagree | 342 (36.8%) | 130 (42.4%) | 0.66 |
| experienced | Neutral | 335 (36.0%) | 86 (28.0%) |  |
| endoscopist | Agree | 253 (27.2%) | 91 (29.6%) |  |
|  |  |  |  |  |
| Support AI in | Disagree | 75 (8.1%) | 18 (5.9%) | 0.14 |
| analysing imagery | Neutral | 167 (17.8%) | 43 (14.0%) |  |
|  | Agree | 688 (74.0%) | 246 (80.1%) |  |
|  |  |  |  |  |
| Physician should | Disagree | 16 (1.7%) | 2 (0.7%) | **0.03** |
| remain responsible | Neutral | 58 (6.2%) | 19 (6.2%) |  |
|  | Agree | 856 (92.0%) | 286 (93.2%) |  |
|  |  |  |  |  |
| Worried by AI | Disagree | 600 (64.5%) | 212 (69.1%) | 0.05 |
| supporting physicians | Neutral | 190 (20.4%) | 70 (22.8%) |  |
|  | Agree | 140 (15.1%) | 25 (8.1%) |  |
|  |  |  |  |  |
| Afraid data fall | Disagree | 431 (46.3%) | 177 (57.7%) | **<0.001** |
| into wrong hands | Neutral | 212 (22.8%) | 67 (21.8%) |  |
|  | Agree | 287 (30.9%) | 63 (20.5%) |  |
|  |  |  |  |  |
| Concerned about | Disagree | 132 (14.2%) | 42 (13.7%) | 0.66 |
| health costs | Neutral | 135 (14.5%) | 39 (12.7%) |  |
|  | Agree | 663 (71.9%) | 226 (73.6%) |  |
|  |  |  |  |  |
| Important AI is cost | Disagree | 31 (3.3%) | 9 (2.9%) | 0.14 |
| effective | Neutral | 161 (17.3%) | 48 (15.6%) |  |
|  | Agree | 738 (79.4%) | 250 (81.4%) |  |
|  |  |  |  |  |
| Concerned about | Disagree | 179 (15.8%) | 1 (23.0%) | 0.27 |
| medical liability | Neutral | 274 (27.2%) | 3 (30.3%) |  |
|  | Agree | 477 (51.3%) | 2 (46.7%) |  |
|  |  |  |  |  |
| Liability for medical | Manufacturer | 166 (17.9%) | 56 (18.2%) | 0.31 |
| error | Endoscopist | 428 (46.0%) | 132 (43.0%) |  |
|  | Hospital | 295 (31.7%) | 96 (31.3%) |  |
|  | Insurance | 26 (2.8%) | 15 (4.9%) |  |
|  | Other | 15 (1.6%) | 8 (2.6%) |  |
|  |  |  |  |  |

*Supplementary Table 4: Comparisons between patients with and without endoscopy experience in patient survey.*

| Variable | Category | No Endoscopy  n (%) | Endoscopy  n (%) | P-value |
| --- | --- | --- | --- | --- |
|  |  |  |  |  |
| Knowledge of AI | Little | 162 (18.1%) | 50 (16.8%) | 0.41 |
|  | Neutral | 388 (43.5%) | 118 (39.6%) |  |
|  | Lot | 343 (38.4%) | 130 (43.6%) |  |
|  |  |  |  |  |
| Trust in AI | Little trust | 186 (20.1%) | 62 (19.8%) | 0.44 |
|  | Neutral | 406 (43.9%) | 130 (41.5%) |  |
|  | Significant | 332 (35.9%) | 121 (38.7%) |  |
|  |  |  |  |  |
| Humans and AI can | Disagree | 75 (8.1%) | 22 (7.0%) | 0.37 |
| complement each | Neutral | 164 (17.8%) | 57 (18.2%) |  |
| other | Agree | 685 (74.1%) | 234 (74.8%) |  |
|  |  |  |  |  |
| Trust use of AI in | Disagree | 142 (15.4%) | 45 (14.4%) | 0.48 |
| endoscopy | Neutral | 281 (30.4%) | 92 (29.4%) |  |
|  | Agree | 501 (54.2%) | 176 (56.2%) |  |
|  |  |  |  |  |
| AI better than | Disagree | 339 (36.7%) | 133 (42.5%) | 0.34 |
| experienced | Neutral | 328 (35.5%) | 93 (29.7%) |  |
| endoscopist | Agree | 257 (27.8%) | 87 (27.8%) |  |
|  |  |  |  |  |
| Support AI in | Disagree | 68 (7.4%) | 25 (8.0%) | 0.76 |
| analysing imagery | Neutral | 164 (17.8%) | 46 (14.7%) |  |
|  | Agree | 692 (74.9%) | 242 (77.3%) |  |
|  |  |  |  |  |
| Physician should | Disagree | 11 (1.2%) | 7 (2.2%) | 0.42 |
| remain responsible | Neutral | 54 (5.8%) | 23 (7.4%) |  |
|  | Agree | 859 (93.0%) | 283 (90.4%) |  |
|  |  |  |  |  |
| Worried by AI | Disagree | 607 (65.7%) | 205 (65.5%) | 0.69 |
| supporting physicians | Neutral | 185 (20.0%) | 75 (24.0%) |  |
|  | Agree | 132 (14.3%) | 33 (10.5%) |  |
|  |  |  |  |  |
| Afraid data fall | Disagree | 427 (46.2%) | 181 (57.8%) | **<0.001** |
| into wrong hands | Neutral | 212 (22.9%) | 67 (21.4%) |  |
|  | Agree | 285 (30.8%) | 65 (20.8%) |  |
|  |  |  |  |  |
| Concerned about | Disagree | 126 (13.6%) | 48 (15.3%) | 0.30 |
| health costs | Neutral | 131 (14.2%) | 43 (13.7%) |  |
|  | Agree | 667 (72.2%) | 222 (70.9%) |  |
|  |  |  |  |  |
| Important AI is cost | Disagree | 27 (2.9%) | 13 (4.2%) | 0.47 |
| effective | Neutral | 159 (17.2%) | 50 (16.0%) |  |
|  | Agree | 738 (79.9%) | 250 (79.9%) |  |
|  |  |  |  |  |
| Concerned about | Disagree | 182 (19.7%) | 65 (20.8%) | 0.11 |
| medical liability | Neutral | 263 (28.5%) | 101 (32.3%) |  |
|  | Agree | 479 (51.8%) | 147 (47.0%) |  |
|  |  |  |  |  |
| Liability for medical | Manufacturer | 164 (17.8%) | 58 (18.5%) | 0.31 |
| error | Endoscopist | 422 (45.7%) | 138 (44.1%) |  |
|  | Hospital | 296 (32.0%) | 95 (30.4%) |  |
|  | Insurance | 29 (3.1%) | 12 (3.8%) |  |
|  | Other | 13 (1.4%) | 10 (3.2%) |  |
|  |  |  |  |  |

*Supplementary Table 5: Comparisons of endoscopist general views on AI by type of endoscopy practice*

| Variable | Category | General  n (%) | Specialist  n (%) | Trainee  n (%) | P-value |
| --- | --- | --- | --- | --- | --- |
|  |  |  |  |  |  |
| Endoscopy | Agree | 157 (92.9%) | 225 (87.6%) | 45 (97.8%) | 0.21 |
| quality improve | Neutral | 11 (6.5%) | 28 (10.9%) | 1 (2.2%) |  |
|  | Disagree | 1 (0.6%) | 4 (1.6%) | 0 (0.0%) |  |
|  |  |  |  |  |  |
| Improved patient | Agree | 146 (86.4%) | 218 (84.8%) | 42 (89.4%) | 0.36 |
| outcome | Neutral | 22 (13.0%) | 34 (13.2%) | 4 (8.5%) |  |
|  | Disagree | 1 (0.6%) | 5 (2.0%) | 1 (2.1%) |  |
|  |  |  |  |  |  |
| Improved | Agree | 145 (86.3%) | 219 (85.6%) | 42 (89.4%) | 0.16 |
| efficiency | Neutral | 18 (10.7%) | 34 (13.3%) | 2 (4.3%) |  |
|  | Disagree | 5 (3.0%) | 3 (1.2%) | 3 (6.4%) |  |
|  |  |  |  |  |  |
| Improve training | Agree | 136 (80.5%) | 204 (79.7%) | 42 (89.4%) | 0.61 |
|  | Neutral | 26 (15.4%) | 38 (14.8%) | 3 (6.4%) |  |
|  | Disagree | 7 (4.1%) | 14 (5.5%) | 2 (4.3%) |  |
|  |  |  |  |  |  |
| Operator | Agree | 62 (36.7%) | 97 (37.7%) | 18 (38.3%) | 0.90 |
| dependence / | Neutral | 64 (37.9%) | 89 (34.6%) | 18 (38.3%) |  |
| de-skilling | Disagree | 43 (25.4%) | 71 (27.6%) | 11 (23.4%) |  |
|  |  |  |  |  |  |
| Financial | Agree | 110 (65.1%) | 203 (79.0%) | 27 (58.7%) | **0.02** |
| reimbursement | Neutral | 54 (32.0%) | 41 (16.0%) | 14 (30.4%) |  |
| important | Disagree | 5 (3.0%) | 13 (5.1%) | 5 (10.9%) |  |
|  |  |  |  |  |  |
| Cost effective | Agree | 89 (53.0%) | 144 (56.3%) | 26 (56.5%) | 0.81 |
|  | Neutral | 57 (33.9%) | 79 (30.9%) | 15 (32.6%) |  |
|  | Disagree | 22 (13.1%) | 33 (12.9%) | 5 (10.9%) |  |
|  |  |  |  |  |  |
| Negatively | Agree | 24 (14.2%) | 31 (12.1%) | 4 (8.7%) | 0.07 |
| impact patient | Neutral | 41 (24.3%) | 60 (23.4%) | 6 (13.0%) |  |
| relationship | Disagree | 104 (61.5%) | 166 (64.6%) | 36 (78.3%) |  |
|  |  |  |  |  |  |
| Concerned about | Agree | 78 (46.2%) | 125 (48.6%) | 21 (45.7%) | 0.66 |
| medical liability | Neutral | 51 (30.2%) | 71 (27.6%) | 7 (15.2%) |  |
|  | Disagree | 40 (23.7%) | 61 (23.7%) | 18 (39.1%) |  |
|  |  |  |  |  |  |
| Liability for | Endoscopist | 96 (56.8%) | 146 (56.8%) | 24 (52.2%) | 0.18 |
| medical error | Hospital | 12 (7.1%) | 18 (7.0%) | 7 (15.2%) |  |
|  | Manufacturer | 43 (25.4%) | 53 (20.6%) | 9 (19.6%) |  |
|  | Insurance | 7 (4.1%) | 7 (2.7%) | 3 (6.5%) |  |
|  | Other | 11 (6.5%) | 33 (12.8%) | 3 (6.5%) |  |
|  |  |  |  |  |  |

*Supplementary Table 6: Comparisons of specific AI applications questions by questions by type of endoscopy practice*

| Variable | Category | General  n (%) | Specialist  n (%) | Trainee  n (%) | P-value |
| --- | --- | --- | --- | --- | --- |
|  |  |  |  |  |  |
| Struggle with | Agree | 101 (59.8%) | 165 (64.2%) | 34 (73.9%) | 0.61 |
| colonic polyp | Neutral | 30 (17.8%) | 40 (15.6%) | 6 (13.0%) |  |
| detection | Disagree | 38 (22.5%) | 52 (20.2%) | 6 (13.0%) |  |
|  |  |  |  |  |  |
| CADe helps | Agree | 146 (86.9%) | 213 (83.2%) | 43 (93.5%) | 0.07 |
| detect clinically | Neutral | 14 (8.3%) | 28 (10.9%) | 2 (4.4%) |  |
| relevant polyps | Disagree | 8 (4.8%) | 15 (5.9%) | 1 (2.2%) |  |
|  |  |  |  |  |  |
| CADe leads to | Agree | 69 (1.1%) | 106 (41.3%) | 17 (37.0%) | 0.79 |
| unnecessary | Neutral | 45 (26.8%) | 66 (25.7%) | 12 (26.1%) |  |
| resections | Disagree | 54 (32.1%) | 85 (33.1%) | 17 (37.0%) |  |
|  |  |  |  |  |  |
| CADe lengthens | Agree | 81 (48.2%) | 127 (49.4%) | 22 (47.8%) | 0.81 |
| procedure times | Neutral | 48 (28.6%) | 58 (22.6%) | 11 (23.9%) |  |
|  | Disagree | 39 (23.2%) | 72 (28.0%) | 13 (28.3%) |  |
|  |  |  |  |  |  |
| Use CADe when | Agree | 143 (85.6%) | 206 (80.2%) | 44 (95.6%) | **0.01** |
| available | Neutral | 24 (14.4%) | 33 (12.8%) | 2 (4.4%) |  |
|  | Disagree | 0 (0.0%) | 18 (7.0%) | 0 (0.0%) |  |
|  |  |  |  |  |  |
| Struggle between | Agree | 102 (60.7%) | 166 (65.6%) | 38 (82.6%) | 0.22 |
| hyperplastics and | Neutral | 37 (22.0%) | 36 (14.0%) | 2 (4.4%) |  |
| adenomas | Disagree | 29 (17.3%) | 55 (21.4%) | 6 (13.0%) |  |
|  |  |  |  |  |  |
| Willing leave | Agree | 105 (62.1%) | 182 (70.8%) | 36 (78.3%) | 0.22 |
| diminutive | Neutral | 28 (16.6%) | 27 (10.5%) | 3 (6.5%) |  |
| rectosig. polyp | Disagree | 36 (21.3%) | 48 (18.7%) | 7 (15.2%) |  |
|  |  |  |  |  |  |
| More comfortable | Agree | 110 (65.1%) | 174 (68.0%) | 35 (76.1%) | 0.66 |
| leaving polyp | Neutral | 37 (21.9%) | 41 (16.0%) | 9 (19.6%) |  |
| if supported by AI | Disagree | 22 (13.0%) | 41 (16.0%) | 2 (4.4%) |  |
|  |  |  |  |  |  |
| Use CADx system | Agree | 150 (89.3%) | 221 (86.0%) | 40 (87.0%) | 0.42 |
| when available | Neutral | 16 (9.5%) | 29 (11.3%) | 5 (10.9%) |  |
|  | Disagree | 2 (1.2%) | 7 (2.7%) | 1 (2.2%) |  |
|  |  |  |  |  |  |
| Struggle with | Agree | 122 (72.2%) | 189 (73.5%) | 39 (84.8%) | 0.17 |
| detection upper GI | Neutral | 22 (13.0%) | 33 (12.8%) | 5 (10.9%) |  |
| neoplasia | Disagree | 25 (14.8%) | 35 (13.6%) | 2 (4.4%) |  |
|  |  |  |  |  |  |
| CADe helpful for | Agree | 144 (85.7%) | 221 (86.0%) | 43 (93.5%) | 0.72 |
| upper GI neoplasia | Neutral | 19 (11.3%) | 31 (12.1%) | 3 (6.5%) |  |
|  | Disagree | 5 (3.0%) | 5 (2.0%) | 0 (0.0%) |  |
|  |  |  |  |  |  |
| CADe leads to | Agree | 67 (39.9%) | 103 (40.1%) | 12 (26.1%) | 0.31 |
| additional biopsies | Neutral | 44 (26.2%) | 75 (29.2%) | 16 (34.8%) |  |
| in upper GI tract | Disagree | 57 (33.4%) | 79 (30.7%) | 18 (39.1%) |  |
|  |  |  |  |  |  |
| Use CADe for GI | Agree | 153 (91.1%) | 222 (86.4%) | 43 (93.5%) | 0.69 |
| neoplasia when | Neutral | 13 (7.7%) | 32 (12.5%) | 3 (6.5%) |  |
| available | Disagree | 2 (1.2%) | 3 (1.2%) | 0 (0.0%) |  |
|  |  |  |  |  |  |

*Supplementary Table 7: Comparisons of endoscopist general views on AI by years of endoscopic experience*

| Variable | Category | 0 – 10 years  n (%) | 11 – 20 years  n (%) | > 20 years  n (%) | P-value |
| --- | --- | --- | --- | --- | --- |
|  |  |  |  |  |  |
| Endoscopy | Agree | 164 (92.7%) | 122 (90.4%) | 143 (88.3%) | 0.25 |
| quality improve | Neutral | 12 (6.8%) | 12 (8.9%) | 16 (9.9%) |  |
|  | Disagree | 1 (0.6%) | 1 (0.7%) | 3 (1.9%) |  |
|  |  |  |  |  |  |
| Improved patient | Agree | 158 (89.3%) | 111 (81.6%) | 139 (85.8%) | 0.16 |
| outcome | Neutral | 16 (9.0%) | 24 (17.7%) | 20 (12.4%) |  |
|  | Disagree | 3 (1.7%) | 1 (0.7%) | 3 (1.9%) |  |
|  |  |  |  |  |  |
| Improved | Agree | 150 (85.7%) | 120 (88.2%) | 138 (85.2%) | 0.83 |
| efficiency | Neutral | 118 (10.3%) | 14 (10.3%) | 22 (13.6%) |  |
|  | Disagree | 7 (4.0%) | 2 (1.5%) | 2 (1.2%) |  |
|  |  |  |  |  |  |
| Improve training | Agree | 140 (79.6%) | 106 (77.9%) | 138 (85.2%) | 0.40 |
|  | Neutral | 25 (14.2%) | 26 (19.1%) | 16 (9.9%) |  |
|  | Disagree | 11 (6.3%) | 4 (2.9%) | 8 (4.9%) |  |
|  |  |  |  |  |  |
| Operator | Agree | 68 (38.4%) | 51 (37.5%) | 58 (35.8%) | 0.50 |
| dependence / | Neutral | 58 (32.8%) | 58 (42.7%) | 57 (35.2%) |  |
| de-skilling | Disagree | 51 (28.8%) | 27 (19.9%) | 47 (29.0%) |  |
|  |  |  |  |  |  |
| Financial | Agree | 118 (66.7%) | 98 (72.6%) | 125 (77.2%) | 0.20 |
| reimbursement | Neutral | 50 (28.3%) | 28 (20.7%) | 31 (19.1%) |  |
| important | Disagree | 9 (5.1%) | 9 (6.7%) | 6 (3.7%) |  |
|  |  |  |  |  |  |
| Cost effective | Agree | 100 (53.0%) | 144 (47.0%) | 98 (60.5%) | 0.18 |
|  | Neutral | 52 (29.6%) | 54 (40.3%) | 45 (27.8%) |  |
|  | Disagree | 24 (13.6%) | 17 (12.7%) | 19 (11.7%) |  |
|  |  |  |  |  |  |
| Negatively | Agree | 22 (12.4%) | 20 (14.8%) | 17 (10.5%) | 0.05 |
| impact patient | Neutral | 27 (15.3%) | 42 (31.1%) | 38 (23.5%) |  |
| relationship | Disagree | 128 (72.3%) | 73 (54.1%) | 107 (66.1%) |  |
|  |  |  |  |  |  |
| Concerned about | Agree | 88 (49.7%) | 60 (44.4%) | 76 (46.9%) | 0.74 |
| medical liability | Neutral | 40 (22.6%) | 43 (31.9%) | 46 (28.4%) |  |
|  | Disagree | 49 (27.7%) | 32 (23.7%) | 40 (24.7%) |  |
|  |  |  |  |  |  |
| Liability for | Endoscopist | 91 (51.4%) | 82 (60.7%) | 94 (58.0%) | 0.22 |
| medical error | Hospital | 19 (10.7%) | 8 (5.9%) | 10 (6.2%) |  |
|  | Manufacturer | 38 (21.5%) | 29 (21.5%) | 38 (23.5%) |  |
|  | Insurance | 8 (4.5%) | 1 (0.7%) | 8 (4.9%) |  |
|  | Other | 21 (11.9%) | 15 (11.1%) | 12 (7.4%) |  |
|  |  |  |  |  |  |

*Supplementary Table 8: Comparisons of specific AI applications questions by years of endoscopic experience*

| Variable | Category | 0 – 10 years  n (%) | 11 – 20 years  n (%) | > 20 years  n (%) | P-value |
| --- | --- | --- | --- | --- | --- |
|  |  |  |  |  |  |
| Struggle with | Agree | 119 (67.2%) | 75 (55.6%) | 108 (66.7%) | **0.04** |
| colonic polyp | Neutral | 22 (12.4%) | 24 (17.8%) | 30 (18.5%) |  |
| detection | Disagree | 36 (20.3%) | 36 (26.7%) | 24 (14.8%) |  |
|  |  |  |  |  |  |
| CADe helps | Agree | 158 (90.3%) | 111 (82.2%) | 135 (83.3%) | **0.008** |
| detect clinically | Neutral | 12 (6.9%) | 15 (11.1%) | 17 (10.5%) |  |
| relevant polyps | Disagree | 5 (2.9%) | 9 (6.7%) | 10 (6.2%) |  |
|  |  |  |  |  |  |
| CADe leads to | Agree | 74 (42.1%) | 51 (37.8%) | 67 (41.4%) | 0.99 |
| unnecessary | Neutral | 41 (23.3%) | 39 (28.9%) | 44 (27.2%) |  |
| resections | Disagree | 61 (34.7%) | 45 (33.3%) | 51 (31.5%) |  |
|  |  |  |  |  |  |
| CADe lengthens | Agree | 81 (46.0%) | 68 (50.4%) | 81 (50.0%) | 0.22 |
| procedure times | Neutral | 37 (21.0%) | 35 (25.9%) | 46 (28.4%) |  |
|  | Disagree | 58 (33.0%) | 32 (23.7%) | 35 (21.6%) |  |
|  |  |  |  |  |  |
| Use CADe when | Agree | 156 (89.1%) | 106 (78.5%) | 133 (82.1%) | **0.002** |
| available | Neutral | 16 (9.1%) | 23 (17.0%) | 20 (12.4%) |  |
|  | Disagree | 3 (1.7%) | 6 (4.4%) | 9 (5.6%) |  |
|  |  |  |  |  |  |
| Struggle between | Agree | 123 (69.9%) | 78 (57.8%) | 107 (66.1%) | 0.05 |
| hyperplastics and | Neutral | 22 (12.5%) | 24 (17.8%) | 29 (17.9%) |  |
| adenomas | Disagree | 31 (17.6%) | 33 (24.4%) | 26 (16.1%) |  |
|  |  |  |  |  |  |
| Willing leave | Agree | 127 (71.8%) | 83 (61.5%) | 114 (70.4%) | 0.06 |
| diminutive | Neutral | 14 (7.9%) | 24 (17.8%) | 20 (12.4%) |  |
| rectosig. polyp | Disagree | 36 (20.3%) | 28 (20.7%) | 28 (17.3%) |  |
|  |  |  |  |  |  |
| More comfortable | Agree | 130 (73.5%) | 87 (64.9%) | 104 (64.2%) | 0.17 |
| leaving polyp | Neutral | 25 (14.1%) | 31 (23.1%) | 31 (19.1%) |  |
| if supported by AI | Disagree | 22 (12.4%) | 16 (11.9%) | 27 (16.7%) |  |
|  |  |  |  |  |  |
| Use CADx system | Agree | 156 (88.6%) | 113 (83.7%) | 144 (88.9%) | **0.04** |
| when available | Neutral | 15 (8.5%) | 20 (14.8%) | 15 (9.3%) |  |
|  | Disagree | 5 (2.8%) | 2 (1.5%) | 3 (1.9%) |  |
|  |  |  |  |  |  |
| Struggle with | Agree | 134 (75.7%) | 98 (72.6%) | 120 (74.1%) | 0.28 |
| detection upper GI | Neutral | 22 (12.4%) | 20 (14.8%) | 18 (11.1%) |  |
| neoplasia | Disagree | 21 (11.9%) | 17 (12.6%) | 24 (14.8%) |  |
|  |  |  |  |  |  |
| CADe helpful for | Agree | 164 (93.2%) | 109 (80.7%) | 137 (84.6%) | **0.003** |
| upper GI neoplasia | Neutral | 8 (4.6%) | 23 (17.0%) | 3 (13.6%) |  |
|  | Disagree | 4 (2.3%) | 3 (2.2%) | 3 (1.9%) |  |
|  |  |  |  |  |  |
| CADe leads to | Agree | 70 (39.8%) | 41 (30.4%) | 72 (44.4%) | 0.17 |
| additional biopsies | Neutral | 40 (22.7%) | 48 (35.6%) | 47 (29.0%) |  |
| in upper GI tract | Disagree | 66 (37.5%) | 46 (34.1%) | 43 (26.5%) |  |
|  |  |  |  |  |  |
| Use CADe for GI | Agree | 167 (91.1%) | 111 (82.2%) | 142 (87.7%) | **0.003** |
| neoplasia when | Neutral | 9 (5.1%) | 24 (17.8%) | 15 (9.3%) |  |
| available | Disagree | 0 (0.0%) | 0 (0.0%) | 5 (3.1%) |  |
|  |  |  |  |  |  |

*Supplementary Table 9: Comparisons of specific AI applications questions between those with and without practical AI experience*

| Variable | Category | No AI experience  n (%) | AI experience  n (%) | P-value |
| --- | --- | --- | --- | --- |
|  |  |  |  |  |
| Endoscopist struggle | Agree | 185 (61.9%) | 120 (66.7%) | 0.37 |
| with colonic polyp | Neutral | 49 (16.7%) | 27 (15.0%) |  |
| detection | Disagree | 63 (21.4%) | 33 (18.3%) |  |
|  |  |  |  |  |
| CADe helps detect | Agree | 250 (85.6%) | 154 (85.6%) | 0.77 |
| clinically relevant polyps | Neutral | 25 (8.6%) | 19 (10.6%) |  |
|  | Disagree | 17 (5.8%) | 7 (3.9%) |  |
|  |  |  |  |  |
| CADe leads to | Agree | 119 (40.6%) | 73 (40.6%) | 0.71 |
| unnecessary resections | Neutral | 75 (25.6%) | 49 (27.2%) |  |
|  | Disagree | 99 (33.8%) | 58 (32.2%) |  |
|  |  |  |  |  |
| CADe lengthens procedure | Agree | 141 (48.1%) | 89 (49.4%) | 0.90 |
| times | Neutral | 80 (27.3%) | 38 (21.1%) |  |
|  | Disagree | 72 (24.6%) | 53 (29.4%) |  |
|  |  |  |  |  |
| Use CADe when available | Agree | 243 (83.2%) | 152 (84.4%) | 0.88 |
|  | Neutral | 38 (13.1%) | 21 (11.7%) |  |
|  | Disagree | 11 (3.8%) | 7 (3.9%) |  |
|  |  |  |  |  |
| Struggle to differentiate | Agree | 187 (63.8%) | 121 (67.2%) | 0.63 |
| between hyperplastics and | Neutral | 50 (17.1%) | 25 (13.9%) |  |
| adenomas | Disagree | 56 (19.1%) | 34 (18.9%) |  |
|  |  |  |  |  |
| Willing leave diminutive | Agree | 198 (67.4%) | 126 (70.0%) | 0.19 |
| rectosigmoid polyps | Neutral | 39 (13.3%) | 19 (10.6%) |  |
|  | Disagree | 57 (19.4%) | 35 (19.4%) |  |
|  |  |  |  |  |
| More comfortable leaving | Agree | 195 (66.3%) | 126 (70.4%) | 0.19 |
| polyp if supported by AI | Neutral | 50 (17.0%) | 37 (20.7%) |  |
|  | Disagree | 49 (1%) | 16 (8.9%) |  |
|  |  |  |  |  |
| Use CADx system when | Agree | 259 (88.4%) | 154 (85.6%) | **0.04** |
| available | Neutral | 29 (9.9%) | 21 (11.7%) |  |
|  | Disagree | 5 (1.7%) | 5 (2.8%) |  |
|  |  |  |  |  |
| Struggle with detection of | Agree | 201 (68.4%) | 151 (83.9%) | **<0.001** |
| upper GI neoplasia | Neutral | 43 (14.6%) | 17 (9.4%) |  |
|  | Disagree | 50 (17.0%) | 12 (6.7%) |  |
|  |  |  |  |  |
| CADe helpful for upper GI | Agree | 254 (86.7%) | 156 (86.7%) | 0.82 |
| neoplasia | Neutral | 32 (10.9%) | 21 (11.7%) |  |
|  | Disagree | 7 (2.4%) | 3 (1.7%) |  |
|  |  |  |  |  |
| CADe leads to additional | Agree | 121 (41.3%) | 62 (34.4%) | 0.87 |
| biopsies in upper GI tract | Neutral | 71 (24.2%) | 64 (35.6%) |  |
|  | Disagree | 101 (34.5%) | 54 (30.0%) |  |
|  |  |  |  |  |
| Use CADe for GI | Agree | 256 (87.4%) | 164 (91.1%) | 0.48 |
| neoplasia when available | Neutral | 35 (12.0%) | 13 (7.2%) |  |
|  | Disagree | 2 (0.7%) | 3 (1.7%) |  |
|  |  |  |  |  |
